# Ylang Ylang Essential Oil in Malignant and Non-Malignant Cells: Comparative Mitophagy-Related Transcriptional Responses

**DOI:** 10.3390/ph19071002

**Published:** 2026-06-28

**Authors:** Goksu Kasarci-Kavsara, Timur Hakan Barak, Baris Ertugrul, Tugba Buse Senturk, Bedia Cakmakoglu, Sinem Bireller

**Affiliations:** 1 Department of Molecular Medicine, Aziz Sancar Institute of Experimental Medicine, Istanbul University, 34093 Istanbul, Türkiye; 2 Department of Pharmacognosy, Faculty of Pharmacy, Acibadem Mehmet Ali Aydinlar University, 34752 Istanbul, Türkiye; 3 Department of Biochemistry, Faculty of Pharmacy, Acibadem Mehmet Ali Aydinlar University, 34752 Istanbul, Türkiye

**Keywords:** Ylang Ylang essential oil, mitochondrial quality control, mitophagy, lung cancer, salivary gland carcinoma, GC-MS-FID

## Abstract

**Background:** Mitophagy is a mitochondrial quality-control pathway whose contribution to cancer stress tolerance may vary by cellular context. For essential oils, mechanistic interpretation is often limited by compositional variability and the limited number of studies addressing malignant and non-malignant comparisons under matched exposure conditions. **Methods:** Ylang Ylang essential oil (YY EO) was characterized by GC-MS-FID. Lung cancer cells (A549) and a salivary gland carcinoma model (HTB-41), together with non-malignant lung-related cells (BEAS-2B, MRC-5), were exposed to YY EO. Functional outcomes were assessed by WST-1 and LDH assays. Mitophagy-related and mitochondrial quality-control-associated genes were quantified by RT-qPCR (2^−ΔΔCt^). **Results:** GC-MS-FID identified a terpenoid-rich mixture (99.31%), with germacrene D and β-caryophyllene among the major constituents. YY EO was associated with dose- and cell-type-dependent functional responses, with malignant cells showing reductions in WST-1 signal and stronger LDH-associated responses under the tested conditions, while non-malignant cells showed less pronounced functional changes. Transcriptional responses were context-dependent, with differential changes in mitophagy-related genes across cell lines. **Conclusions:** These findings provide comparative evidence of greater functional sensitivity in malignant cells, alongside cell-context-dependent mitophagy-related transcriptional responses. These observations are hypothesis-generating and remain limited to functional readouts and mRNA-level data. Within these limits, the present study provides a composition-anchored comparative dataset that may support future mechanistic studies in this area.

## 1. Introduction

**Figure 1:**
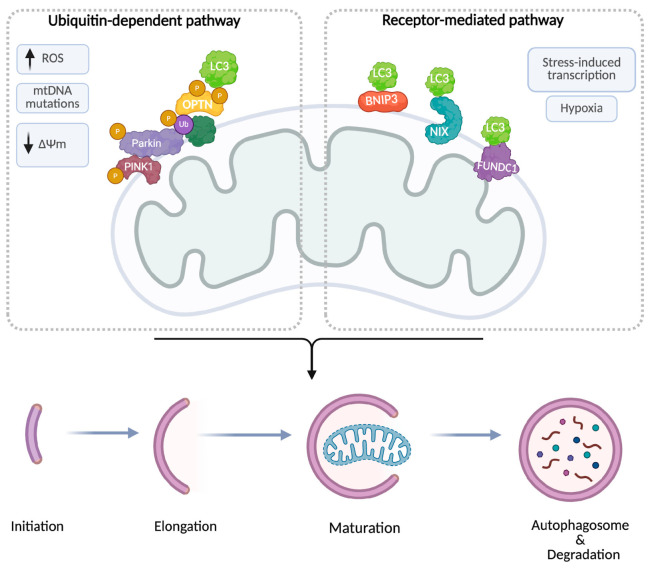
Schematic overview of mitophagy, illustrating mitochondrial damage sensing, ubiquitin-dependent (PINK1/PRKN) and receptor-mediated (BNIP3, NIX, and FUNDC1) mitophagy pathways, including recruitment of autophagy adaptors (OPTN, NDP52, p62), and LC3-mediated autophagosome formation. Dotted boxes indicate the two major mitophagy pathways, arrows indicate the direction of process progression, and the lower bracket highlights their convergence into the shared autophagosome formation and degradation sequence (created in BioRender; Bireller, S. (2026)).

Mitochondria sustain cellular viability through the integration of ATP production, metabolic signaling, and stress adaptation. Beyond energy supply, they also function as signaling hubs that shape inflammatory tone and cell-fate decisions under stress, making mitochondrial quality-control pathways disproportionately influential in disease contexts. Mitophagy is an evolutionarily conserved quality-control pathway that removes dysfunctional mitochondria to preserve organelle integrity [1,2,3]. It is typically engaged through two routes, the ubiquitin-mediated PINK1/PARKIN and receptor-mediated (BNIP3, NIX, FUNDC1) pathways, that recruit mitochondria to the autophagic machinery via LC3-interacting motifs (Figure 1) [4,5,6]. These routes rely on LC3 to translate mitochondrial damage sensing into selective autophagic clearance.

Mitophagy proceeds through recognizable phases, from damage sensing to lysosomal degradation, and turns out to be particularly relevant under oxidative or metabolic stress [5,7]. When this clearance system is impaired, mitochondrial dysfunction, ROS accumulation, and inflammatory signaling may escalate, linking defective mitophagy to multiple pathologies, including malignancy and fibrotic lung disorders [3,4,5,8]. Conversely, under persistent stress, mitophagy may support survival by preserving and maintaining mitochondrial fitness and limiting bioenergetic collapse. This bidirectional behavior supports viewing mitophagy as a stress adaptation program whose net effect depends on stress intensity, duration, and cellular metabolic state.

Recent work increasingly describes mitophagy as a route-diverse, threshold-sensitive mitochondrial stress response shaped by cell type, metabolic state, and exposure dynamics. This perspective highlights a challenge for multi-component exposures. Adaptive mitochondrial quality-control engagement and overt bioenergetic failure may lead to partially overlapping mitophagy-related outcomes under marker-limited or non-matched designs. Essential oils (EOs) are well positioned in this context with their widely reported redox-modulating and antioxidant-associated bioactivities with mitochondria-related stress responses. These properties make mitochondrial quality-control pathways a plausible common mechanistic layer through which EOs might influence cellular outcomes. Nevertheless, compositional uncertainty and the relative scarcity of side-by-side malignant and non-malignant comparisons under standardized exposure conditions continue to constrain mechanistic attribution and claims of differential functional sensitivity. In the present study, GC–MS–FID verification of Ylang Ylang essential oil (YY EO) was integrated with complementary functional readouts and targeted RT-qPCR profiling of mitophagy and mitochondrial quality-control genes (BNIP3, NIX, LC3, FUNDC1, PINK1/PARKIN, LONP1, ATP5F1A, SRC) to enable a composition-anchored study of context-dependent mitochondrial stress in malignant models (lung cancer and salivary gland carcinoma models) and non-malignant lung counterparts.

In cancer biology, mitophagy is often described as a context-dependent regulator. Defective mitophagy may permit damaged mitochondria to persist, promoting excess ROS and genomic instability that may support malignant transformation; however, adaptive mitophagy may also help malignant cells tolerate hypoxia, nutrient limitations, and therapy-associated stress [9,10]. Consistent with this duality, mitophagy-related proteins (PINK1, PARKIN, BNIP3, NIX) are frequently dysregulated in lung cancer particularly in NSCLC and have been linked with both tumor-suppressive effects and resistance phenotypes [9,10,11,12,13]. As a result, targeting mitochondrial quality control presents a fundamental selectivity challenge, requiring disruption of tumor stress tolerance while preserving cellular viability in non-malignant cells. The selectivity problem is particularly relevant for multi-component interventions, where dose and exposure time may shift cells between adaptive and injurious stress thresholds. Although therapeutic options have expanded for lung cancer, many interventions remain limited in their ability to reverse tissue damage. In NSCLC, especially lung adenocarcinoma, intracellular alterations frequently include mitochondrial impairment and disturbance of the immune microenvironment [12,13].

Comparable mitochondrial stress programs are increasingly discussed across head-and-neck malignancies, including salivary gland malignancies and other head and neck cancers, although this literature is less mature than that of lung cancer. In this context, mitophagy has been linked to apoptosis and senescence-like phenotypes as well as invasive behavior in salivary gland carcinoma models, highlighting a potentially actionable mitochondrial quality-control pathway [14,15,16]. As a result, many patients require aggressive multimodal therapy, yet recurrence and treatment resistance remain central limitations. Mitophagy is also a candidate pathway supporting tolerance to hypoxia, nutrient limitation, and therapy-associated pressure. The mitochondria and mitophagy pathways are increasingly viewed as determinants of treatment responses rather than passive bystanders [9,11]. However, composition-anchored studies that compare malignant and non-malignant cells side-by-side under matched exposure conditions remain relatively limited in salivary gland carcinoma models. This gap is methodologically important, as the absence of matched exposure comparisons allows claims of selective vulnerability to be confounded by baseline growth rates, metabolic state, or differential stress-buffering capacity.

Despite advances in multimodal treatment strategies (surgery, chemotherapy, radiotherapy, targeted therapy and immunotherapy), recurrence and treatment resistance remain major clinical barriers in both lung cancer and salivary gland malignancies [17,18]. With mitochondria governing apoptotic priming, metabolic flexibility, and redox balance, mitochondrial dynamics and mitophagy are repeatedly implicated in tumor stress tolerance and therapy adaptation [4,6,8,9]. Mitophagy-related programs may support drug-tolerant persistent states and influence treatment responsiveness in a context-dependent manner [10,12]. From a translational perspective, this suggests that a useful intervention might not simply increase or decrease mitophagy; instead, it may alter mitochondrial stress responses in a manner that depends on cellular context and exposure conditions.

Within this frame, natural products have attracted attention as multi-target bioactive sources capable of influencing mitochondrial stress responses [19,20,21]. Essential oils (EOs) are volatile, terpenoid-rich mixtures with broadly reported bioactivities (antioxidant, anti-inflammatory, antimicrobial), and their multi-component nature supports evaluation as modulators of redox and stress-related pathways [20,21,22]. These mechanistic insights support a shift from empirical use toward evidence-based applications [21,23]. A composition-anchored design remains particularly important, given that the differences in dominant terpenoids and minor constituents may reshape biological response profiles [20,21].

YY EO, derived from *Cananga odorata* flowers, is commercially important and has been associated with antioxidant and broader bioactive properties [20,23,24,25,26,27]. Direct cell-based evidence focused specifically on YY EO remains limited, and cancer-related studies have recently begun to accumulate. The available literature includes broader evaluations of *Cananga odorata* bioactivity [27,28,29], formulation-based work using YY EO in triple-negative breast cancer models [30], and more recent keratinocyte-based studies suggesting that formulation might influence on biological readouts [31]. Overall, these reports indicate a growing, but still limited, body of experimental studies on YY EO in defined cellular systems. They also show that the available evidence remains heterogeneous in terms of formulation strategy, exposure design, and biological endpoints. In view of potential variability in YY EO composition, compositional verification is critical for mechanistic interpretation; therefore, GC–MS–FID profiling of the YY EO used in this study was combined with functional and transcriptional assessment of mitophagy-related pathways. Genes related to mitophagy and mitochondrial quality-control pathways and stress adaptation (BNIP3, NIX, LC3, FUNDC1, PINK1/PARKIN, LONP1, ATP5F1A, SRC) were examined in lung and salivary gland carcinoma models, alongside non-malignant counterparts, to understand whether YY EO influences mitochondrial quality-control responses in a context-dependent manner [2,5,19,21,32]. The study examined whether composition-verified YY EO might be associated with different functional and transcriptional responses in malignant and non-malignant cells under matched exposure conditions. To our knowledge, comparative data directly examining chemically characterized YY EO in malignant and non-malignant cell models under the same tested conditions remain limited. The present study was designed as a preliminary comparative evaluation combining functional assays with transcriptional analysis of selected mitophagy-related and mitochondrial quality-control-associated genes.

The central premise of this work is that YY EO may influence mitochondrial stress handling and mitophagy-related signaling in a context-dependent manner [20,22,23,30,33,34,35]. In this sense, mitochondrial stress handling refers to the balance between redox load, bioenergetic capacity, and quality-control engagement, rather than a simple activation or inhibition of mitophagy. We therefore hypothesized that composition-verified YY EO might be associated with differential mitochondrial stress responses in malignant and non-malignant cells. This design enabled a composition-anchored, side-by-side assessment of whether YY EO is associated with greater functional sensitivity in malignant cells, alongside comparatively limited functional changes in non-malignant cells under the tested conditions.

## 2. Results

### 2.1. Phytochemical Investigation

Detailed GC-MS-FID analysis was conducted on the YY EO, and results are given in Table 1. A total of 99.31% of the sample was revealed and 42 different compounds were identified. β-caryophyllene and germacrene D were determined as the major ingredients with contents of 15.26 ± 0.04 and 19.25 ± 0.03%, respectively. In addition, α-farnesene and linalool were calculated in significant amounts, 8.43 ± 0.04 and 7.64 ± 0.22%, respectively. The results revealed the complex nature of the EO with molecules from various groups such as monoterpenes, sesquiterpenes, and hydrocarbons. These findings establish a foundational basis for elucidating the potential bioactivities of the sample and may guide future investigations into its therapeutic applications.

### 2.2. The Effect of Ylang Ylang Essential Oil on WST-1-Based Functional Responses

**Figure 2:**
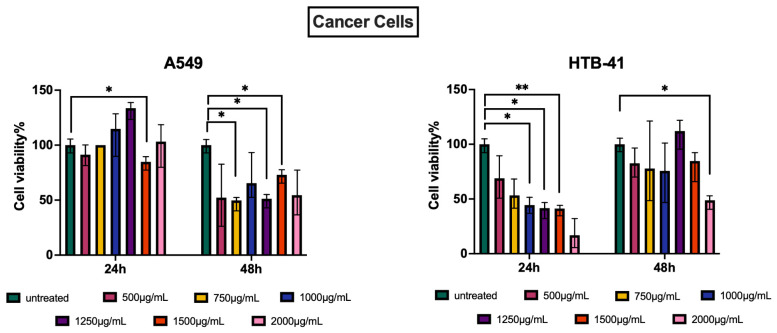
Effects of YY EO on cell viability in malignant cell models (A549 and HTB-41) at 24 h and 48 h, assessed by WST-1 assay. Cells were treated with increasing concentrations of YY EO (500–2000 μg/mL). Data are presented as mean ± SD from three independent experiments. * *p* < 0.05 and ** *p* < 0.01 compared with the untreated (control) group.

**Figure 3:**
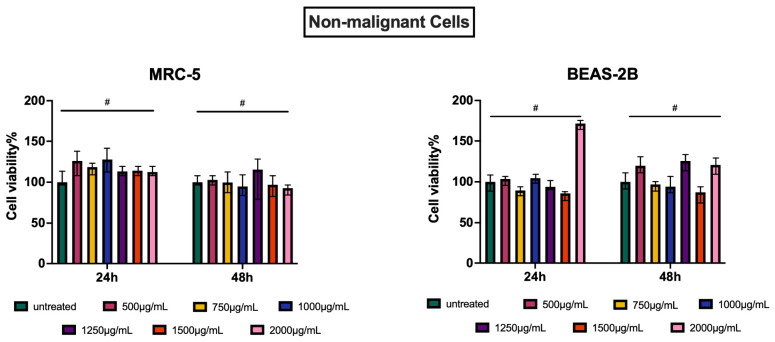
Effects of YY EO on cell viability in non-malignant cell models (MRC-5 and BEAS-2B) at 24 h and 48 h, assessed by WST-1 assay. Cells were treated with increasing concentrations of YY EO (500–2000 μg/mL). Data are presented as mean ± SD from three independent experiments. *#* indicates comparison with the untreated (control) group (*p* < 0.05).

The effect of YY EO across the tested concentration range (500–2000 µg/mL) was initially assessed using the WST-1 assay as an indirect functional readout at different time points. Cells were exposed to the tested concentration range for 24 and 48 h to identify concentration and time windows suitable for downstream comparative analyses. As shown in Figure 2 and Figure 3, YY EO treatment was associated with statistically significant, time- and dose- dependent reductions in WST-1 signal in A549 and HTB-41 cells compared with untreated controls. In contrast, no comparable decrease in WST-1 signal was observed in non-malignant lung cell models. In MRC-5 and BEAS-2B cells, WST-1 values generally remained above 80% under the tested conditions and, in some settings, were slightly higher than control values. These findings indicate a differential response between malignant and non-malignant cells under the tested conditions. Because WST-1 is an indirect assay, these signal changes were interpreted cautiously and were not taken as direct evidence of a specific mechanism.

Based on initial results, 1250 µg/mL and 1500 µg/mL were selected as working concentrations for downstream analyses in both A549 and HTB-41 cell lines. These concentrations were selected from the initial screening as working concentrations, because they produced measurable but incomplete WST-1 signal reduction in the malignant cell models and allowed subsequent LDH and RT-qPCR analyses to be performed under the same conditions. Based on WST-1 outcomes, 48 h was selected for A549 cells and 24 h for HTB-41 cells. Because the present study was designed to define comparative working concentrations for matched downstream analyses rather than to establish potency rankings, the selected dose framework was carried forward on this basis. At these time points, A549 cells showed 51.2% and 73.01% relative WST-1 signals, whereas HTB-41 cells exhibited 41.6% and 41.2%, respectively, at 1250 µg/mL and 1500 µg/mL. Notably, A549 did not show a strictly concentration-dependent response across the selected working concentrations, suggesting that the response pattern under these conditions was not fully linear.

Unlike malignant cell lines, no significant decrease in WST-1 signal was observed in the non-malignant counterparts MRC-5 and BEAS-2B. In both non-malignant models, WST-1 values remained above 80% at both concentrations and time points, and, for some conditions, showed a slight increase relative to controls. Taken together, these findings suggest greater functional sensitivity in the malignant cell models under the tested exposure conditions. However, WST-1 alone does not distinguish between reduced viability, reduced proliferation, altered metabolic state, or other stress-associated changes. For this reason, the WST-1 findings were interpreted together with the LDH results and should be regarded as indirect functional observations under the tested conditions.

### 2.3. The Effect of Ylang Ylang Essential Oil on LDH-Associated Membrane Integrity Responses

To further examine whether the YY EO-associated functional changes were accompanied by altered membrane integrity, LDH release was evaluated as a complementary readout in the selected cell models (Table 2). The effect of YY EO was studied at concentrations of 1250 µg/mL and 1500 µg/mL, which had been selected as working concentrations based on the WST-1 screening results, in both non-malignant and malignant cell lines. In the present study, LDH results are reported as LDH-derived cytotoxicity (%) calculated by low- and high-control normalization and additionally expressed as membrane integrity (%) = 100 − cytotoxicity (%), where lower membrane integrity indicates greater membrane compromise.

**Figure 4:**
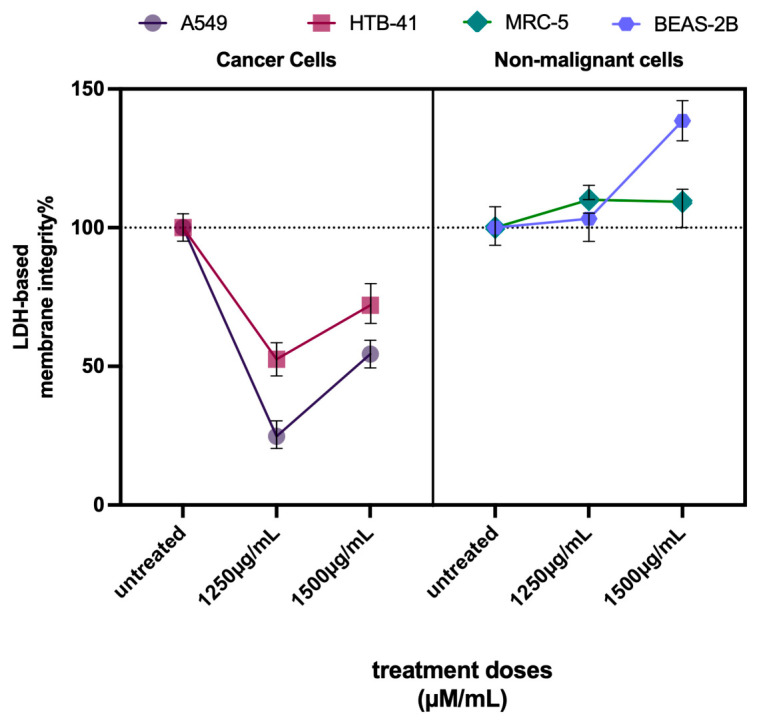
Membrane integrity assessment following YY EO exposure, quantified by LDH release in A549, HTB-41, MRC-5, and BEAS-2B cell lines at the indicated concentrations. Data are presented as mean ± SD from three independent experiments (*p* < 0.05). LDH-derived membrane integrity (%) was calculated as 100 − cytotoxicity (%). Higher values indicate better membrane integrity (lower membrane damage). The dotted horizontal line marks the 100% membrane integrity reference level corresponding to the untreated control. Statistical comparisons were performed relative to untreated controls. (Values below 0% or above 100% were capped at 0 and 100%, respectively).

To assess membrane integrity under YY EO exposure, LDH release was measured following YY EO treatment in the cell lines. Results are expressed as LDH-derived cytotoxicity (%) and normalized to untreated control groups. In the A549 cell line, treatment with 1250 µg/mL and 1500 µg/mL YY EO resulted in cytotoxicity values of 24.8% and 54%, respectively. For the HTB-41 cell line, the corresponding values were 52.57% and 71.99% (Figure 4).

By contrast, in non-malignant cell lines BEAS-2B and MRC-5, LDH values remained close to baseline or showed only slight increases relative to controls, indicating comparatively preserved membrane integrity under the tested conditions (Figure 4). LDH release reflects membrane damage but does not define the mode of cell death. Therefore, these findings were interpreted as complementary functional evidence of differential membrane response under the tested conditions rather than as direct evidence of a specific death mechanism. Taken together, the combined WST-1 and LDH findings suggest greater functional sensitivity in the malignant cell models under the tested conditions.

### 2.4. Regulation of Gene Expression Associated with the Mitophagy-Related Pathways in Response to Ylang Ylang Essential Oil

**Figure 5:**
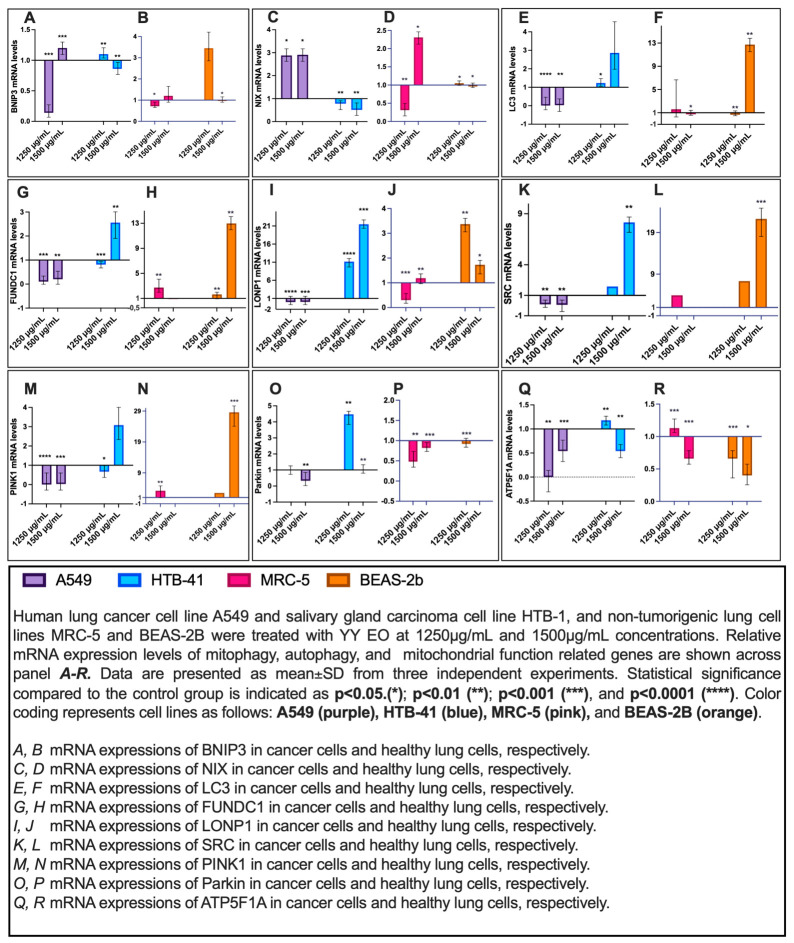
Effects of YY EO treatment on the mRNA expression of mitophagy-, autophagy-, and mitochondrial-function-related genes in cancer and non-malignant cell lines. Relative mRNA expression levels of BNIP3, BNIP3L (NIX), LC3, FUNDC1, LONP1, SRC, PINK1, PARKIN, and ATP5F1A are shown across panels (**A**–**R**). Human lung cancer cell lines A549 and HTB-41, and non-malignant lung cell lines MRC-5 and BEAS-2B, were treated with YY EO at 1250 and 1500 µg/mL. Gene expression levels were quantified by RT-qPCR and normalized to the untreated control group using the 2^−ΔΔCt^ method. The dotted horizontal line indicates the control expression level (set to 1). Data are presented as mean ± SD from three independent experiments. Statistical significance compared with the control group is indicated as *p* < 0.05 (*), *p* < 0.01 (**), *p* < 0.001 (***), and *p* < 0.0001 (****).

To explore the mitochondrial response to YY EO, gene expression profiling was conducted using RT-qPCR. Genes involved in mitophagy and mitochondrial quality control—including BNIP3, NIX, LC3, FUNDC1, LONP1, ATP5F1A, and PINK1/PARKIN—were analyzed in both malignant and non-malignant cell lines. As shown in Figure 5A–R, YY EO treatment was associated with differential changes in the expression of these genes in malignant cells and non-malignant cells in a cell type- and dose-dependent manner. Because these data are transcriptional, they are interpreted as pathway-engagement changes rather than direct measures of mitophagy flux.

#### 2.4.1. Malignant Cell Lines Show Distinct Transcriptional Changes in Mitophagy- and Mitochondrial-Stress-Related Genes Following YY EO Exposure

In A549 cells, YY EO treatment was associated with an overall downregulation of several mitophagy- and mitochondrial-function-related genes. Notably, BNIP3 expression exhibited a dose-dependent biphasic response, with significant suppression observed at 1250 µg/mL, followed by a modest increase at 1500 µg/mL YY EO (Figure 5A). In parallel, NIX gene expression was significantly increased at both YY EO concentrations (2.88- and 2.91-fold, respectively, *p* < 0.05) compared with the control group (Figure 5C). In addition, PINK1 and PARKIN expression levels showed a slight but consistent decrease following YY EO treatment (Figure 5M,O), consistent with comparatively limited transcriptional change in the PINK1/PARKIN pathway in A549 cells. Alongside these changes, YY EO treatment also resulted in a consistent suppression of remaining mitophagy- and mitochondrial-stress-associated genes, including FUNDC1, LC3, ATP5F1A, and SRC, collectively reflecting a broad transcriptional downregulation of mitochondrial quality-control components in A549 cells.

In HTB-41 cells (Figure 5-blue bars), YY EO treatment elicited a broader response compared with A549 cells (Figure 5-purple bars). At the lower YY EO concentration, HTB-41 cells showed a partial transcriptional response in mitophagy-related genes. Receptor-mediated mitophagy markers were largely unchanged to slightly decreased, with modest reductions in NIX and FUNDC1 and only a mild increase in BNIP3 (Figure 5A,C,G). In contrast, LONP1, PARKIN, and SRC were significantly upregulated at both 1250 µg/mL and 1500 µg/mL YY EO concentrations. Specifically, LONP1 expression increased by 11.17-fold and 21.47-fold, PARKIN by 4.47-fold and 1.03-fold, and SRC by 1.87-fold and 8.15-fold, respectively (*p* < 0.05) (Figure 5I,O,K). Exposure to the higher YY EO concentration was associated with a more coordinated transcriptional pattern in HTB-41 cells. Under these conditions, FUNDC1 expression was strongly induced, whereas BNIP3 and NIX remained at low levels, a pattern consistent with the relative transcriptional prominence of FUNDC1-associated signaling in the higher-exposure condition.

#### 2.4.2. Non-Malignant Lung Cell Lines Display Heterogeneous Mitochondrial- and Mitophagy-Related Transcriptional Responses to YY EO

In MRC-5 lung fibroblasts, YY EO exposure resulted in relatively modest alterations in mitophagy- and mitochondrial quality-control-related genes. At both concentrations, receptor-mediated mitophagy markers BNIP3, NIX, and FUNDC1 showed mild but statistically significant changes, without a pronounced dose-dependent amplification (Figure 5B,D,H). LC3 and PINK1 showed only limited responses, primarily at 1500 µg/mL of YY EO, whereas PARKIN levels remained close to control values (Figure 5N,P). Notably, ATP5F1A was slightly reduced, consistent with subtle transcriptional adjustments in mitochondrial energy metabolism without clear induction of mitophagy-related pathways. In contrast, BEAS-2B bronchial epithelial cells showed a more pronounced, dose-dependent response to YY EO. LC3, FUNDC1, LONP1, SRC, PINK1 and PARKIN were significantly upregulated, particularly at 1500 µg/mL, indicating coordinated transcriptional upregulation of both receptor-mediated and PINK1/PARKIN-associated mitochondrial quality-control components (Figure 5F,H,J,L,N,P). Concurrently, ATP5F1A expression was markedly decreased, suggesting a transcriptional shift away from oxidative phosphorylation under higher YY EO exposure (Figure 5R).

## 3. Discussion

Natural products constitute a major source of lead compounds and novel therapeutics, encompassing constituents and metabolites derived from plants, animals, insects, and microorganisms, as well as a broad range of endogenous chemical components [36]. Among plant-derived natural products, essential oils represent mixtures of volatile secondary metabolites, primarily composed of terpenoids and related hydrocarbons [37,38,39]. By anchoring biological effects to a verified chemical profile and comparing malignant and non-malignant cells under matched exposure conditions, this study aimed to distinguish differences in functional sensitivity from mitochondrial quality-control-related transcriptional changes, outcomes that are often conflated in essential oil literature. Due to their chemically diverse composition, essential oils have attracted increasing interest for their broad spectrum of biological activities, including antimicrobial, anti-inflammatory, antioxidant, and anticancer effects [38,39]. In this study, GC–MS–FID compositional verification of YY EO was integrated with functional readouts and gene-expression profiling of mitophagy- and mitochondrial quality-control-related pathways to examine a composition-anchored response pattern under the tested conditions.

Overall, the results showed a differential response under the tested conditions. YY EO was associated with lower WST-1 signal and reduced membrane integrity in the malignant cell models, whereas the non-malignant counterparts showed comparatively limited changes in these functional readouts. WST-1 signals decreased in A549 and HTB-41 in a time-dependent manner, whereas MRC-5 and BEAS-2B generally maintained high WST-1 values under matched exposure conditions (Figure 2 and Figure 3). LDH-based membrane integrity measurements showed the same general pattern, with greater membrane compromise in the malignant cells and comparatively limited changes in the non-malignant cells (Figure 4). Taken together, the findings suggest greater functional sensitivity in the malignant cell models under the tested exposure conditions. However, given that WST-1 and LDH are indirect functional assays, these observations should not be taken as direct evidence of a specific mechanism or mode of cell death.

A recurring limitation in essential oil studies is compositional variability across geography, cultivation, and distillation parameters. Therefore, mechanistic interpretation requires composition-anchored studies. In line with this requirement, our phytochemical profiling confirmed that the YY EO used here is a terpenoid-rich, multi-component matrix (Table 1), allowing the biological findings to be interpreted in relation to the tested preparation rather than to YY EO in general [20,22,24,25,27].

YY EO is used for various purposes, including therapeutic applications as well as in the food and cosmetics industries [33]. Thus, it has high commercial value; multiple producer countries exist, and it is an important export material [26]. However, there are multiple types of YY EOs available on the market. Samples are available in five different grades, Extra, I, II, III, and Complete, and with increasing distillation time there is an increase in the less volatile fraction of sesquiterpenes and a decrease in highly volatile compounds. The Complete grade is a mixture of all grades obtained in fractional distillation and is highly popular in use [25]. Therefore, for this study, the Complete grade YY EO was used. There are various studies in the literature analyzing phytochemical profiles of YY EO samples from different sources, showing significant variation due to geography, production, climate, and other factors [24]. Thus, it is crucial to evaluate the phytochemical profile of the samples prior to biological activity studies to discuss possible mechanisms of action.

In our study, 99.31% of the sample was identified and results are reported in Table 1. Germacrene D and β-caryophyllene were the top two major constituents, consistent with the compositional ranges reported across multiple YY EO studies. For example, Ng et al. (2021) reported marked variation between origins, emphasizing the importance of phytochemical verification even within the same plant species [24]. Similarly, Lebanov et al. (2020) analyzed 18 different YY EO samples and reported wide ranges for β-caryophyllene and germacrene D, alongside substantial variation in other abundant constituents [25]. Taken together, these reports reinforce that composition anchoring is essential for mechanistic interpretation. In addition, previously reported inconsistencies in product quality for oils marketed as “pure” further underline the need for chromatographic verification prior to bioactivity claims being made [40].

Beyond confirming the importance of GC-MS-FID verification for commercially sourced YY EO, our compositional analysis also provides context for discussing the possible biological relevance of major constituents identified in the tested preparation, particularly β-caryophyllene and germacrene D. GC/MS-based studies have repeatedly identified β-caryophyllene as one of the major sesquiterpene constituents across a broad range of essential oils, and β-caryophyllene-containing profiles have been associated with diverse biological activities, including antimicrobial and anticancer-related effects [37,39,41]. Given reports linking β-caryophyllene to mitochondrial membrane integrity and oxidative stress signaling, and the recurrent association of germacrene D with cytotoxic EO profiles, it is plausible that these abundant constituents contribute to the observed phenotype, although the present data support a mixture-driven effect rather than attribution to a single component. Several reports suggest that β-caryophyllene may modulate mitochondrial membrane integrity, oxidative stress signaling, and intrinsic stress-responsive pathways that intersect with mitochondrial quality-control and mitophagy [42,43]. In addition to β-caryophyllene, germacrene D has been associated with cytotoxic and stress-related cellular responses in essential oil studies employing compositional profiling and functional bioassays [44]; however, evidence directly supporting germacrene D as the primary active component remains limited. In line with these reports, our GC-MS-FID analysis identified β-caryophyllene and germacrene D among the predominant components of YY EO, leading us to consider the possibility that these constituents might contribute, at least in part, to the functional and transcriptional response pattern observed in the present study. Taken together, these reports support the possibility that abundant constituents such as β-caryophyllene and germacrene D might contribute to the observed response pattern. However, the present study was not designed to assign the observed effects to individual constituents. Accordingly, the current findings are more appropriately interpreted as reflecting the activity of the tested YY EO mixture rather than the effect of a single dominant component.

The difference between malignant and non-malignant responses should be interpreted with caution. Under the tested conditions, malignant cells showed a greater reduction in WST-1 signal together with a greater loss of membrane integrity, whereas the non-malignant cells retained comparatively stable functional readouts. This pattern is consistent with greater functional sensitivity in the malignant models; however, firmer conclusions regarding mechanism, selectivity, or direct cell-death pathways would require additional validation [45]. Modest increases in WST-1 signal in some non-malignant conditions were not interpreted as evidence of a defined adaptive mechanism, since WST-1 alone does not distinguish among altered metabolic state, proliferation-related changes, oxidative stress-associated responses, or assay-specific effects.

Our study investigated the effects of YY EO on mitophagy- and mitochondrial quality-control-related pathways in malignant and non-malignant cells. Previous studies have demonstrated that mitophagy can be initiated through both receptor-mediated mechanisms involving BNIP3, NIX, and FUNDC1, and ubiquitin-dependent pathways mediated by PINK1 and PARKIN, thereby regulating mitochondrial homeostasis under cellular stress conditions [4,6,46]. In our results, YY EO did not induce a uniform, directional transcriptional change across all lines (Figure 5). Instead, mitophagy- and mitochondrial quality-control-related transcripts showed cell-type- and dose-dependent differences, supporting the view that YY EO exposure was associated with transcriptional changes in these pathways under the tested conditions. Importantly, because the present analysis was limited to mRNA-level data, these findings should not be interpreted as direct evidence of mitophagy flux or pathway activation at the protein level [5,7].

In A549 cells, YY EO was associated with broad downregulation across multiple mitochondrial quality-control-related genes, while showing a notable increase in NIX (~2.9-fold at both doses) and a biphasic BNIP3 response (Figure 5). This pattern suggests that YY EO exposure was associated with a non-uniform transcriptional response in A549 cells rather than with a consistent shift across all mitophagy-related markers. In studies evaluating clinical tumor specimens in NSCLC, increased BNIP3 expression has been reported in tumors, with high BNIP3 levels associated with poorer prognosis in specific cohorts [47,48]. At the protein level, cytoplasmic BNIP3 expression has been reported to correlate with HIF-1 and other hypoxia markers, suggesting a potential role for BNIP3 in hypoxia-associated stress adaptation [47]. Against this background, the transcriptional differences observed here might be discussed in relation to stress-associated mitochondrial signaling; however, this interpretation remains preliminary.

HTB-41 cells showed a more dynamic transcriptional response. At 1250 µg/mL, the receptor-mediated route appeared partially suppressed (NIX and FUNDC1 slightly reduced, BNIP3 mildly increased), while LONP1, PARKIN, and SRC were significantly upregulated at both doses. At 1500 µg/mL, FUNDC1 induction became more pronounced while BNIP3/NIX remained low, accompanied by LC3/LONP1 increases and decreased ATP5F1A, indicating a more stressed transcriptional profile in the higher-exposure condition. In this salivary gland carcinoma model, YY EO was associated with broader transcriptional changes than those observed in A549 cells. FUNDC1-LC3 binding is regulated by the FUNDC1 LIR motif and by phosphorylation at Ser13, Ser17, and Tyr18; under hypoxia or mitochondrial stress, Ser13/Tyr18 dephosphorylation has been linked to enhanced LC3 binding [49]. The increase in PINK1 expression at this dose is compatible with mitochondrial-stress-related signaling, which may accompany mitochondrial depolarization or protein import stress [50]. The concurrent upregulation of LC3 and LONP1 supports the presence of mitochondrial quality-control-related transcriptional changes; however, mitophagy flux cannot be inferred from mRNA changes alone, and flux assays rely on LC3 dynamics together with mitochondrial protein turnover [48]. Conversely, the reduction in ATP5F1A expression suggested a decline in OXPHOS capacity at the higher YY EO concentration.

Non-malignant cell lines also diverged from each other, underscoring that healthy responses are not uniform. MRC-5 remained comparatively stable, with PARKIN expression remaining close to that of the control and only subtle shifts in energy-related transcription. BEAS-2B showed a coordinated and dose-dependent response. At 1500 µg/mL, LC3, FUNDC1, LONP1, SRC, PINK1, and PARKIN increased together while ATP5F1A decreased. This contrast suggests that the non-malignant response was also cell-type-dependent, rather than uniform across both models.

Despite advances in multimodal treatment strategies, recurrence and resistance remain major clinical barriers in lung and salivary gland malignancies. Mitochondria govern apoptotic priming, metabolic flexibility, and redox balance; thus, mitochondrial dynamics and mitophagy are repeatedly implicated in chemo-resistant phenotypes [4,8]. In this context, the present study provides a preliminary, composition-anchored comparison of YY EO-associated functional and transcriptional responses in malignant and non-malignant cell models. Although the findings do not establish mechanism or therapeutic selectivity, they may provide a useful basis for future mechanistic studies and follow-up experimental work in this area.

### Limitations

Several limitations warrant consideration. First, transcriptional changes do not quantify mitophagy flux; future work should integrate protein-level and flux-based assays, together with mitochondrial morphology and functional readouts, to clarify pathway directionality. Accordingly, the changes observed in the present study should be interpreted as mitophagy-related transcriptional responses rather than as direct evidence of pathway activation or autophagic turnover. Second, batch-to-batch compositional verification and standardization are important for reproducibility. The present study was conducted using a single commercially obtained YY EO preparation. Although this preparation was chemically characterized by GC-MS-FID, essential-oil composition may vary according to source, batch, distillation profile, storage conditions, and commercial origin. Therefore, the current findings should be interpreted within the limits of the tested preparation and should not be generalized to all YY EO samples. In addition, the functional assays used in this study, including WST-1 and LDH, provide indirect readouts and do not by themselves distinguish among altered metabolic state, proliferation-related effects, oxidative stress-associated responses, or specific modes of cell death. Finally, broader dose- and time-dependent studies across additional cell types and incorporating combination designs with standard chemotherapeutics would help determine whether YY EO influences sensitivity or resistance-related response patterns in more clinically relevant strategies. Isolated-constituent testing, reconstituted-mixture experiments, and broader cross-batch validation were beyond the scope of the present study. Within these limits, the present work should be regarded as a preliminary composition-anchored dataset that may support future mechanistic and reproducibility-focused studies.

## 4. Materials and Methods

### 4.1. Cell Models

Human lung carcinoma epithelial-like cells (A549; ATCC, Manassas, VA, USA) and human submaxillary salivary gland carcinoma cells (HTB-41; ATCC, Manassas, VA, USA) were used as malignant models. Non-malignant lung cell lines included human bronchial epithelial cells (BEAS-2B; ATCC, Manassas, VA, USA) and human lung fibroblasts (MRC-5; ATCC, Manassas, VA, USA) (Table 2). Cells were maintained under standard culture conditions (37 °C, 5% CO_2_) in the recommended media supplemented with fetal bovine serum and antibiotics as per routine practice.

### 4.2. GC-MS-FID Analysis of Ylang Ylang Essential Oil

YY EO (*Cananga odorata*) was commercially purchased from a pharmacy store in Istanbul, Türkiye. To preserve experimental consistency and eliminate inter-batch chemotypic variation, a single production batch was utilized for all parallel experiments. The essential oil was stored in its original airtight amber glass vial and maintained in a dark, climate-controlled environment (20 °C ± 2) throughout the study. To characterize the phytochemical composition of the *Cananga odorata* (Ylang Ylang) essential oil used in the present study, a comprehensive Gas Chromatography–Mass Spectrometry coupled with Flame Ionization Detection (GC–MS-FID) analysis was performed. The essential oil was obtained commercially and diluted in hexane prior to injection. GC-MS-FID was performed using an HP-5MS column (30 m × 0.25 mm) (Agilent technologies 7890 A GC system, Santa Clara, CA, USA), with helium as the carrier gas at a constant flow rate of 0.9 mL/min. The oven temperature was initially set at 60 °C for 1 min, ramped to 246 °C at a rate of 3 °C/min, and then held constant for 30 min. A 1 μL volume of the sample was injected in split mode (25:1). At the end of the separation, analytes were directed to both MS and FID detectors via the splitter simultaneously. Compound identification was based on comparison of calculated retention indices (relative to an n-alkane series) with literature data, as well as spectral matching using the NIST17 mass spectral library. Relative percentages of the identified compounds were calculated based on peak area integration from the FID chromatograms and reference substances were used where applicable [51].

### 4.3. Cell Treatment and Concentration Selection for Ylang Ylang Essential Oil

To determine the working concentration range of YY EO, cells were exposed to increasing concentrations of YY EO for varying durations. Initial screening was performed using the WST-1 assay as an indirect functional readout, and based on these findings, LDH assays were subsequently conducted to evaluate membrane integrity at selected working concentrations of YY EO.

#### 4.3.1. Cell Viability Assessment: WST-1 Assay

Cells listed in Table 2 were cultured under standard protocols and conditions were seeded at 1 × 10^4^ cell/well in 96-well plates and treated with increasing concentrations of YY EO (ranging from 500 µg/mL to 2000 µg/mL) for 24 and 48 h. YY EO was first dissolved in DMSO, and all experimental groups (including controls) received the same final DMSO concentration (vehicle control; final DMSO: 0.1% (*v*/*v*)). Vehicle controls were included at each time point. Following incubation, 10 µL of WST-1 (Sigma Aldrich, St. Louis, MO, USA) reagent was added to each well, and plates were incubated at 37 °C for 4 h. Absorbance was measured at 440 nm to quantify WST-1 reduction as a tetrazolium-reduction-based functional readout. To control for possible assay interference from the EO matrix, cell-free wells containing YY EO at each concentration in complete medium were processed in parallel during WST-1 measurements. Cell responses are expressed relative to the untreated control group. Based on these screening results, working concentrations and exposure times were selected for downstream analyses.

#### 4.3.2. Evaluation of Membrane Integrity: LDH Assay

The release of Lactate Dehydrogenase (LDH), a stable cytosolic enzyme, was used as an indicator of cell membrane damage for all cells (Table 2). Following treatment with the previously selected working YY EO concentrations, culture supernatants were collected, and membrane damage was assessed using an LDH cytotoxicity colorimetric assay kit (Elabscience, Houston, TX, USA) according to the manufacturer’s instructions. Vehicle-treated wells were used as low-control wells, YY EO-treated wells were used as sample wells, and high-control wells were prepared according to the kit protocol. After the incubation and centrifugation steps, supernatants were transferred to a fresh plate, the reaction working solution was added, and absorbance was measured at 450 nm with a reference wavelength of 600 nm using a microplate reader (Thermo Fisher Scientific, Waltham, MA, USA). LDH outcomes are reported as LDH-derived cytotoxicity (%) calculated using low- and high-control normalization and are additionally expressed as membrane integrity (%) = 100 − cytotoxicity (%). Medium-only wells were included for background subtraction. The final DMSO concentration (0.1% (*v*/*v*)) was kept constant across all groups.

### 4.4. Mitochondrial Gene Expression Profiling via qPCR

#### 4.4.1. RNA Extraction and cDNA Synthesis

Following treatment, total RNA was isolated using a commercial Invitrogen PureLink RNA Mini kit (Thermo Fisher Scientific, Waltham, MA, USA). Purity and concentration were assessed with a NanoDrop spectrophotometer (Thermo Fisher Scientific, Waltham, MA, USA). RNA integrity was confirmed by the 260/280 nm absorbance ratio. Subsequently, cDNA was synthesized using 200 ng of RNA per reaction. RNA was mixed with oligo(dT) 18 primers (Table A1) and incubated at 65 °C for 5 min, followed by addition of reverse transcription reagents including 5× buffer, dNTPs, M-MuLV reverse transcriptase, and RNase inhibitor. The reaction proceeded at 42 °C for 60 min and was terminated at 99 °C for 5 min.

#### 4.4.2. Real-Time PCR

Quantitative gene expression analysis was performed using SYBR Green-based Real-Time PCR on a LightCycler^®^ 96 Instrument (Roche Diagnostics, Mannheim, Germany). Primers targeted genes associated with mitochondrial function, oxidative stress, mitophagy, and respiratory complex assembly (including BNIP3, LC3, LONP1, FUNDC1, SRC, ATP5F1A, PINK1, PARKIN). Primer sequences and melting temperatures are provided in Appendix A Table A1. Gene expression was normalized to GAPDH, and relative expression was calculated using the 2^−ΔΔCt^ method.

### 4.5. Statistical Analysis

Statistical analyses were conducted using GraphPad Prism version 10 (GraphPad Software, Boston, MA, USA). Data are presented as mean ± standard deviation (SD) from three independent biological experiments, each performed with technical triplicates. Vehicle controls containing the same final DMSO concentration were included at each time point. Relative gene expression was calculated using the comparative Ct method (2^−ΔΔCt^) with GAPDH as the reference gene. Normality was assessed using the Shapiro–Wilk test. For normally distributed data, one-way ANOVA followed by Dunnett’s multiple comparisons test was used to compare each YY EO treatment group with the corresponding vehicle control within each cell line. No separate outlier-exclusion step was applied during data analysis. A *p*-value < 0.05 was considered statistically significant. Statistical significance is indicated as *p* < 0.05 (*), *p* < 0.01 (**), *p* < 0.001 (***), and *p* < 0.0001 (****).

## 5. Conclusions

In conclusion, by anchoring YY EO to its GC-MS-FID composition and integrating functional readouts with mitophagy-related transcriptional changes, this study provides comparative preliminary evidence that YY EO was associated with greater functional sensitivity in lung and salivary gland carcinoma models than in non-malignant counterparts under the tested exposure conditions. These findings support a differential response pattern between malignant and non-malignant cells under the tested conditions. However, the present data remain limited to indirect functional readouts and mRNA-level measurements. Accordingly, they should be regarded as hypothesis-generating and do not establish direct mitophagy flux, pathway directionality, or definitive cell-death selectivity. Further studies incorporating protein-level analyses, flux-based assays, and broader standardization of YY EO composition will be needed to clarify the mechanistic basis of these observations and their potential relevance in combination-oriented or translational settings. In addition, because the study was conducted using a single chemically characterized YY EO preparation, the findings should be interpreted within the limits of the tested composition.

## Figures and Tables

**Table 1:** Phytochemical analysis results of YY EO with GC-MS-FID.

R.T	RRI Exp.	RRI Lit.	Compounds	%	IM
8.63	932	932	α-Pinene	0.19 ± 0.00	RI, MS, RS
10.46	985	977	β-Pinene	0.06 ± 0.00	RI, MS
10.58	988	992	β-Myrcene	0.06 ± 0.00	RI, MS, RS
11.84	1021	1021	*p*-Cresyl methyl ether	2.12 ± 0.21	RI, MS
12.20	1030	1033	Eucalyptol	0.25 ± 0.05	RI, MS, RS
14.96	1100	1096	Methyl benzoate	0.28 ± 0.01	RI, MS
15.22	1106	1104	Linalool	7.64 ± 0.22	RI, MS, RS
17.94	1169	1163	Benzyl Acetate	0.96 ± 0.03	RI, MS
19.08	1195	1195	α-Terpineol	0.08 ± 0.01	RI, MS, RS
19.31	1201	1202	Estragole	0.12 ± 0.01	RI, MS
21.90	1260	1258	Geraniol	1.12 ± 0.03	RI, MS, RS
25.31	1340	1350	α-Cubebene	0.13 ± 0.00	RI, MS
25.82	1352	1363	Eugenol	0.18 ± 0.00	RI, MS, RS
26.43	1367	1372	Ylangene	0.55 ± 0.07	RI, MS
27.02	1380	1377	α-Copaene	1.16 ± 0.05	RI, MS
27.42	1391	1389	Geranyl acetate	7.31 ± 0.01	RI, MS, RS
27.70	1397	1391	β-Elemene	0.68 ± 0.01	RI, MS
29.09	1432	1426	β-Caryophyllene	15.26 ± 0.04	RI, MS, RS
29.24	1435	1433	β-Copaene	0.59 ± 0.01	RI, MS
29.82	1450	1441	Aromadendrene	0.15 ± 0.00	RI, MS
30.09	1456	1439	Isogermacrene D	0.35 ± 0.00	RI, MS
30.33	1462	1458	Humulene	4.50 ± 0.03	RI, MS
30.52	1467	1463	*cis*-β-farnesene	0.12 ± 0.01	RI, MS
30.62	1469	1470	Bicyclosesquiphellandrene	0.28 ± 0.01	RI, MS
31.59	1494	1486	Germacrene D	19.25 ± 0.03	RI, MS
31.69	1496	1491	γ-Amorphene	1.11 ± 0.01	RI, MS
31.88	1501	1498	Bicyclogermacrene	1.10 ± 0.03	RI, MS
32.09	1507	1502	α-Muurolene	1.83 ± 0.05	RI, MS
32.26	1511	1498	α-Bergamotene	2.37 ± 0.01	RI, MS
32.46	1516	1511	α-Farnesene	8.43 ± 0.04	RI, MS
33.09	1533	1524	δ-Cadinene	4.21 ± 0.01	RI, MS
34.42	1567	1556	Elemol	0.12 ± 0.01	RI, MS
35.61	1599	1587	Caryophyllene oxide	0.08 ± 0.01	RI, MS, RS
36.62	1626	1622	Junenol	0.35 ± 0.00	RI, MS
37.09	1639	1625	1-*epi*-Cubenol	0.14 ± 0.00	RI, MS
37.51	1650	1640	τ-Cadinol	1.53 ± 0.00	RI, MS
37.65	1655	1652	Muurolol	0.44 ± 0.00	RI, MS
38.01	1664	1654	α-Cadinol	2.25 ± 0.27	RI, MS
40.29	1728	1749	Farnesol	2.01 ± 0.09	RI, MS
42.09	1780	1770	Benzyl benzoate	6.76 ± 0.02	RI, MS
44.31	1846	1846	Farnesyl acetate	1.73 ± 0.01	RI, MS
45.46	1881	1862	Benzyl salicylate	1.46 ± 0.01	RI, MS
			Total identified	99.31	

R.T: retention time. RRI exp.: linear program retention indices determined in this study. RRI lit.: retention indices from the literature. IM: identification method. RI: retention index, MS: mass Library, RS: reference substance.

**Table 2:** List and descriptive characteristics of the cell lines.

Cell Models	Type of Definition
Cancer Cell Lines	
CCL-185 (A549)	Human lung carcinoma epithelial-like cell
HTB-41 (A-253)	Human submaxillary salivary gland carcinoma cell
Non-malignant (healthy) cell lines	
BEAS-2B (CRL-9609)	Human bronchial epithelial cell
MRC-5 (CCL-171)	Human lung fibroblast cell line

## Data Availability

The raw data supporting the conclusions of this article will be made available by the authors on request.

## Appendix A

Supplementary methodological details, including gene-specific primer sequences and melting temperatures used for RT-qPCR analysis, are summarized in Table A1.

**Table A1:** Gene-specific primer sequences and melting temperatures.

	Primer ID	Sequence (5′→3′)	Tm (°C)
Receptor-mediated mitophagy pathway	*BNIP3*	F: CAGGGCTCCTGGGTAGAACT R: CTACTCCGTCCAGACTCATGC	56
	*BNIP3L (NIX)*	F: TCCACTTCAGACACCCTAAACGT R: TTTCCCTCAGTAGGTGCTGGCA	54
	*MAP1LC3a (LC3)*	F: GCTACAAGGGTGAGAAGCAGCT R: CTGGTTCACCAGCAGGAAGAAG	54
	*FUNDC1*	F: TAATGGGTGGCGTTACTGGC R: AGCCACTATGACTAGCAATCTGA	59
Ubiquitin-mediated mitophagy pathway	*PINK1*	F: AGAGCCACAGTGACAGATCC R: GTGGTGGCTAGTGCTCCTAC	54
	*PARKIN*	F: GTGCAGAGACCGTGGAGAAA R: GCTGCACTGTACCCTGAGTT	59
Mitophagy-associated regulatory genes	*LONP1*	F: TGGAGGAAGACCACTACGGCAT R: GCCATAGAAGCAGAGGATCTTGC	54
	*SRC*	F: TCCTCGTGCGAGAAAGTGAG R: CTGCTCATTCCACAACACCC	54
	*ATP5F1A*	F: ATGACGACTTATCCAAACAGGC R: CGGGAGTGTAGGTAGAACACAT	56
Internal control	*GAPDH*	F: GAAGGTGAAGGTCGGAGTCAA R: TGACAAGCTTCCCGTTCTCAG	56
